# Supplementary material for: Insights on improving accessibility and usability of functional data to unlock its potential for variant interpretation
Source: medRxiv. 2025 Jan 27:2025.01.25.25321117. Preprint. [Version 1] doi: 10.1101/2025.01.25.25321117 (PMC11838987; doi:10.1101/2025.01.25.25321117)

medRxiv preprint doi: https://doi.org/10.1101/2025.01.25.25321117; this version posted January 27, 2025. The copyright holder for this preprint (which was not certified by peer review) is the author/funder, who has granted medRxiv a license to display the preprint in perpetuity.
It is made available under a CC-BY 4.0 International license .

Supplemental Figures

Figure S1. Exploration of variant interpretation practice by respondents. (A) Approximate number of variants interpreted by respondents per year divided into five groups (None, 1-5 variants, 6-25 variants, 26-100 variants, more than 100 variants). **(B)** Approximate number of VUS reinterpreted by respondents per year divided into five groups (None, 1-5 variants, 6-25 variants, 26-100 variants, more than 100 variants). **(C)** Proportion of VUS updated after reinterpretation by respondents per year. **(D)** Proportion of VUS classification that were due to insufficient data. **(F)** Proportion of VUS that had functional data available for use in variant interpretation. Scales ranging from “Never (0 times)” to “Very Frequently (8-10 times out of 10)” were used for Figure C, D, and E.

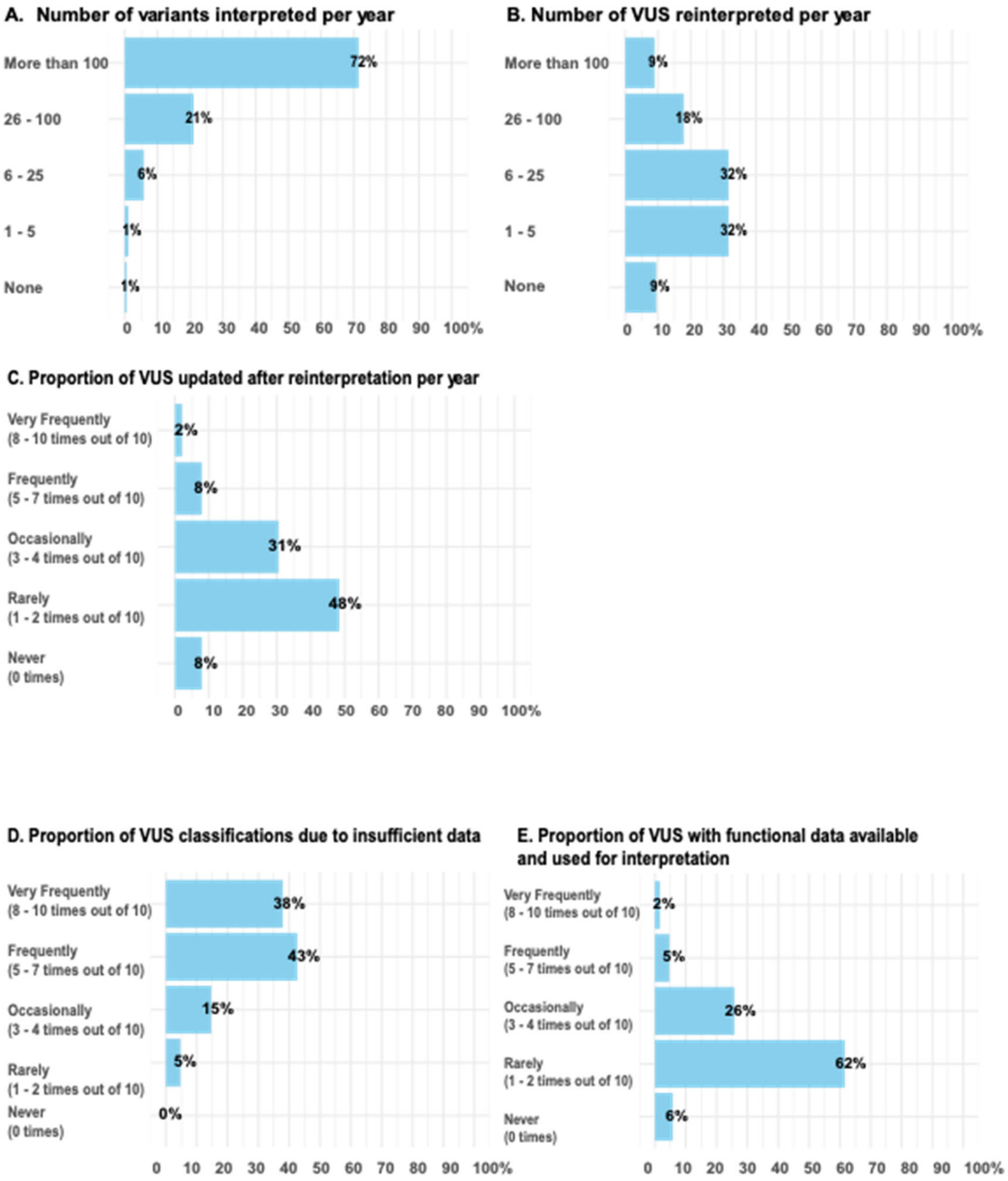

medRxiv preprint doi: https://doi.org/10.1101/2025.01.25.25321117; this version posted January 27, 2025. The copyright holder for this preprint (which was not certified by peer review) is the author/funder, who has granted medRxiv a license to display the preprint in perpetuity.
It is made available under a CC-BY 4.0 International license .

Figure S2. High-throughput functional assay as least comfortable functional evidence type across years of experience. Bar charts showing different types of functional assays ranked based on the comfort level score given by the respondents stratified by years of experience. Responses that gave the same score for all assays were excluded from this analysis. None of the responses were missing value for all five assays/models. Total n = 160 (0-5 years: n = 44, 6-10 years: n = 37, 11-20 years: n = 50, More than 20: n = 26).

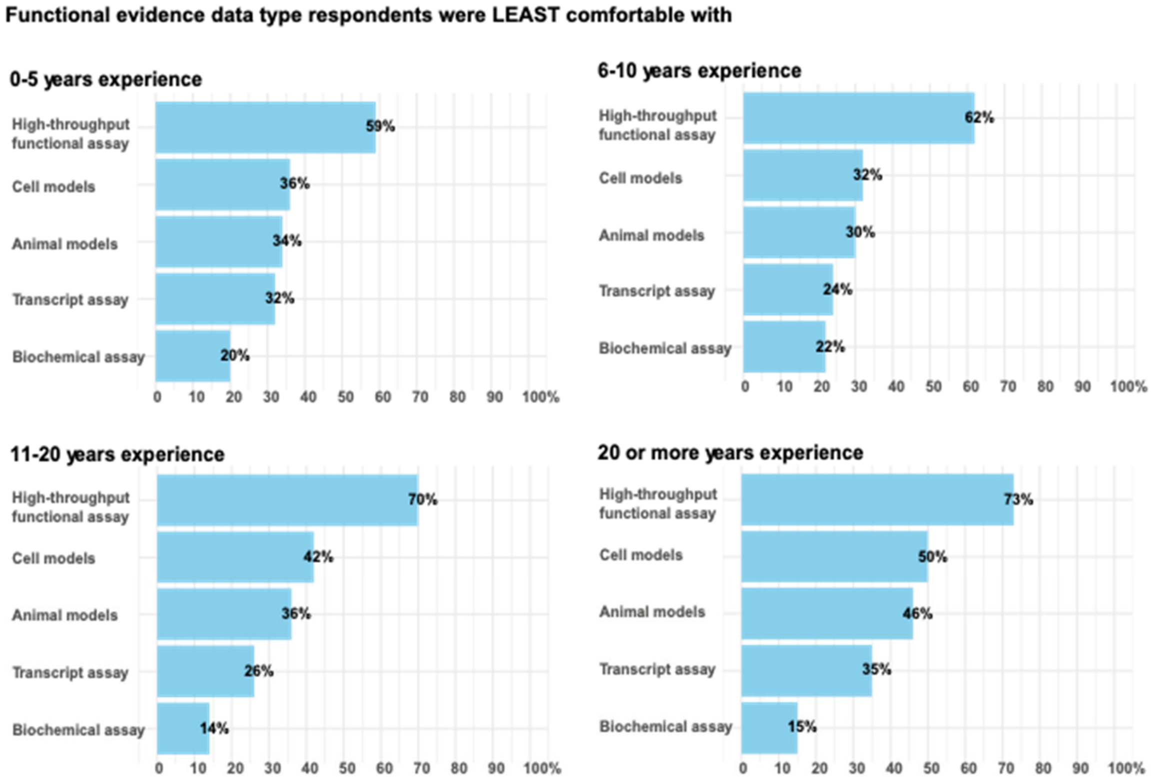

medRxiv preprint doi: https://doi.org/10.1101/2025.01.25.25321117; this version posted January 27, 2025. The copyright holder for this preprint (which was not certified by peer review) is the author/funder, who has granted medRxiv a license to display the preprint in perpetuity.
It is made available under a CC-BY 4.0 International license .

Figure S3. Respondents' comfort level and awareness of various functional evidence use/assessment guidelines and resources. (A) Respondents' comfort level and awareness of various functional evidence guidelines. (**B)** Respondents' comfort level and awareness of various functional evidence resources. N = 190. Non-responses are not shown but were less than 2% for all items.

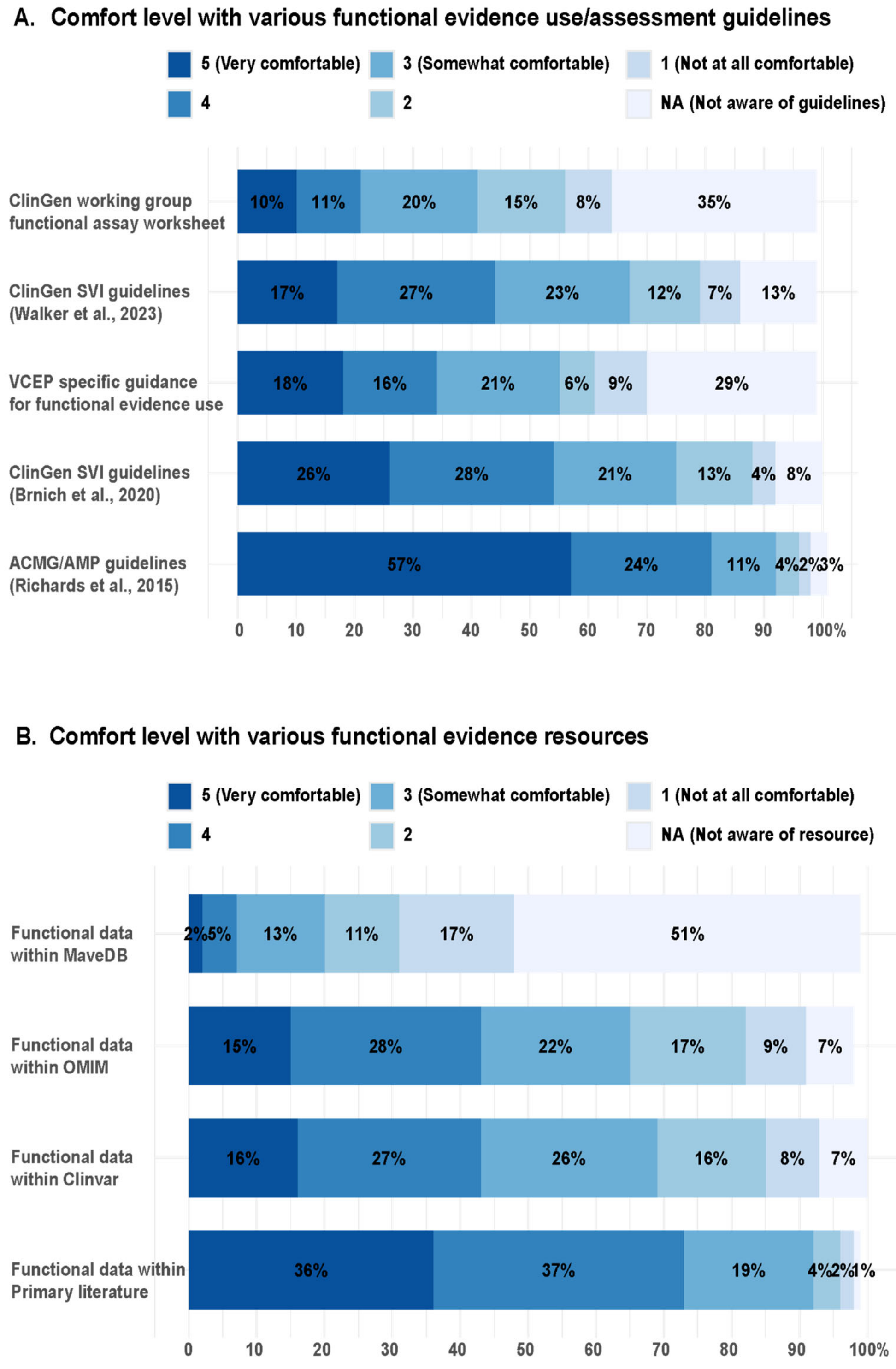

Supplement: Supplement 1 [file NIHPP2025.01.25.25321117v1-supplement-1.pdf]
